# Post-cardiac Injury Syndrome Following Catheter Ablation of Atrial Flutter: A Case Report

**DOI:** 10.7759/cureus.67735

**Published:** 2024-08-25

**Authors:** Md Abidur Reza Chowdhury, Mohamed Ebrahim Mostafa, Ahmed Attia, Gasim Mohammedahmed

**Affiliations:** 1 Cardiology, University Hospitals of Leicester NHS Trust, Leicester, GBR

**Keywords:** atrial flutter, catheter ablation, pericarditis, dressler syndrome, post cardiac injury syndrome

## Abstract

A woman in her 70s presented to accident and emergency (A&E) with shortness of breath and fever following atrial flutter ablation. Initial investigations revealed a new onset of pleural and pericardial effusion with raised inflammatory markers. After systematically ruling out chest infection and heart failure, a diagnosis of post-cardiac injury syndrome (PCIS) was made. After a short course of steroids and colchicine, she showed significant improvement in her symptoms, and subsequent follow-up showed resolution of her pleural and pericardial effusion.

## Introduction

The term post-cardiac injury syndrome (PCIS) was initially introduced to describe pleuro-pericarditis following myocardial infarction (Dressler’s syndrome) or cardiac surgery (post-pericardiotomy syndrome) [1]. However, PCIS can complicate percutaneous coronary intervention [2], pacemaker implantation [3], and cardiac catheterization [4]. The pathophysiology of PCIS is not well understood. It is suggested to be an immune-mediated inflammatory syndrome secondary to injury to the pericardium, myocardium, pleura, and even lung tissues [5,6]. The immune-mediated pathogenesis is supported by a latent period generally of a few weeks until the appearance of the first manifestation and response to anti-inflammatory drugs.

PCIS after catheter ablation has been less commonly reported. For example, Magnocavallo et al. reported 0.04% incidence of PCIS following atrial fibrillation ablation [7]. Due to its rarity, PCIS can be too late to be diagnosed and presents a challenge to physicians. Therefore, an open-minded approach and a low threshold for diagnosis are necessary for early diagnosis and prompt management.

## Case presentation

A woman in her 70s presented to accident and emergency (A&E) with complaints of recent onset of shortness of breath. It had been going on for three days and was associated with fever and generalized weakness. She mentioned shortness of breath at mild to moderate exertion and denied any orthopnea or paroxysmal nocturnal dyspnea. She had an atrial flutter ablation two weeks back with no complication, and during that time, a post-procedure echo ruled out any pericardial effusion or tamponade. The patient denied any chest pain, cough, or palpitation during this presentation. Her past medical history included hypertension and a history of bowel cancer (under remission). On examination, she was tachycardic, and chest auscultation revealed diminished bilateral breath sound. However, there was no audible pericardial rub. Heart rate was 100 beats per minute (regular), and blood pressure was 110/67 mmHg. She also spiked a temperature of 38°C during admission.

**Figure 1:**
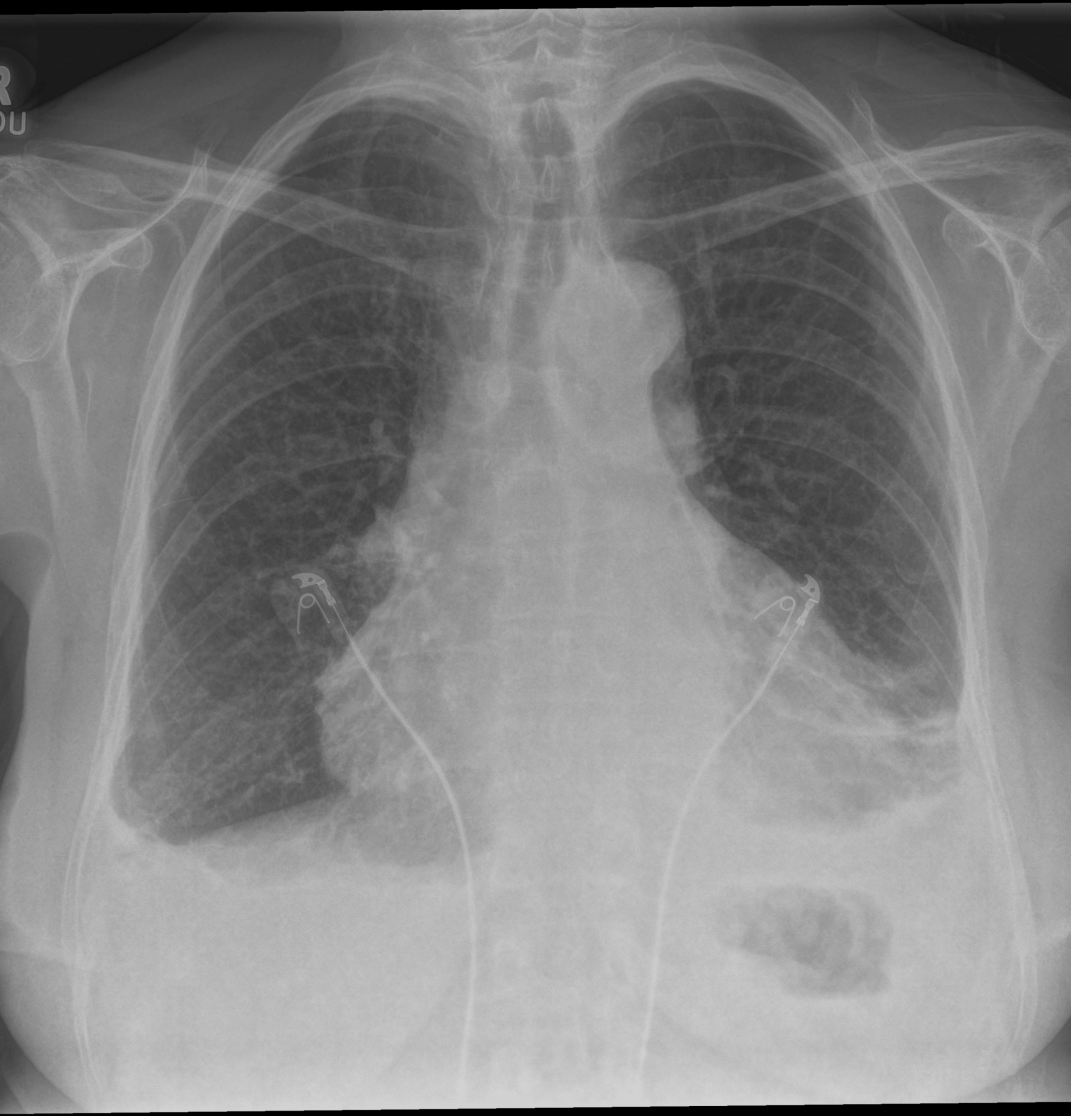
Chest X-ray showing bilateral pleural effusion

**Figure 2:**
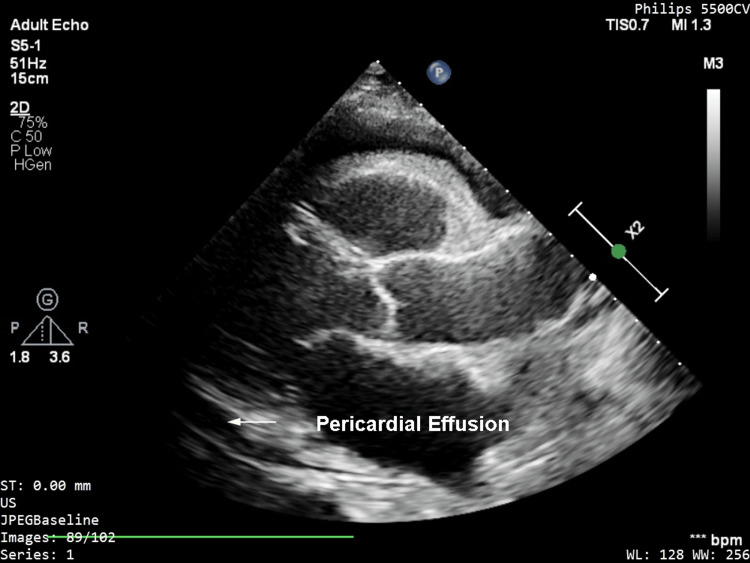
Transthoracic echocardiography showing moderate-size pericardial effusion

Initial investigations showed elevated white cell counts (14000/mm^3^) with predominant neutrophilia, and hemoglobin was within normal limits; however, C-reactive protein (CRP) was raised (258 mg/L). The rest of the blood, including troponin, NT-proBNP, urea, and electrolytes, were within normal limits. ECG showed sinus tachycardia without any signs of pericarditis. The chest X-ray showed bilateral pleural effusion (Figure 1), and bedside echocardiography revealed moderate global pericardial effusion of 1.2 cm (Figure 2) without any signs of tamponade. Left ventricular systolic function was normal on visual estimation. There was no evidence of elevated left ventricular filling pressure.

**Figure 3:**
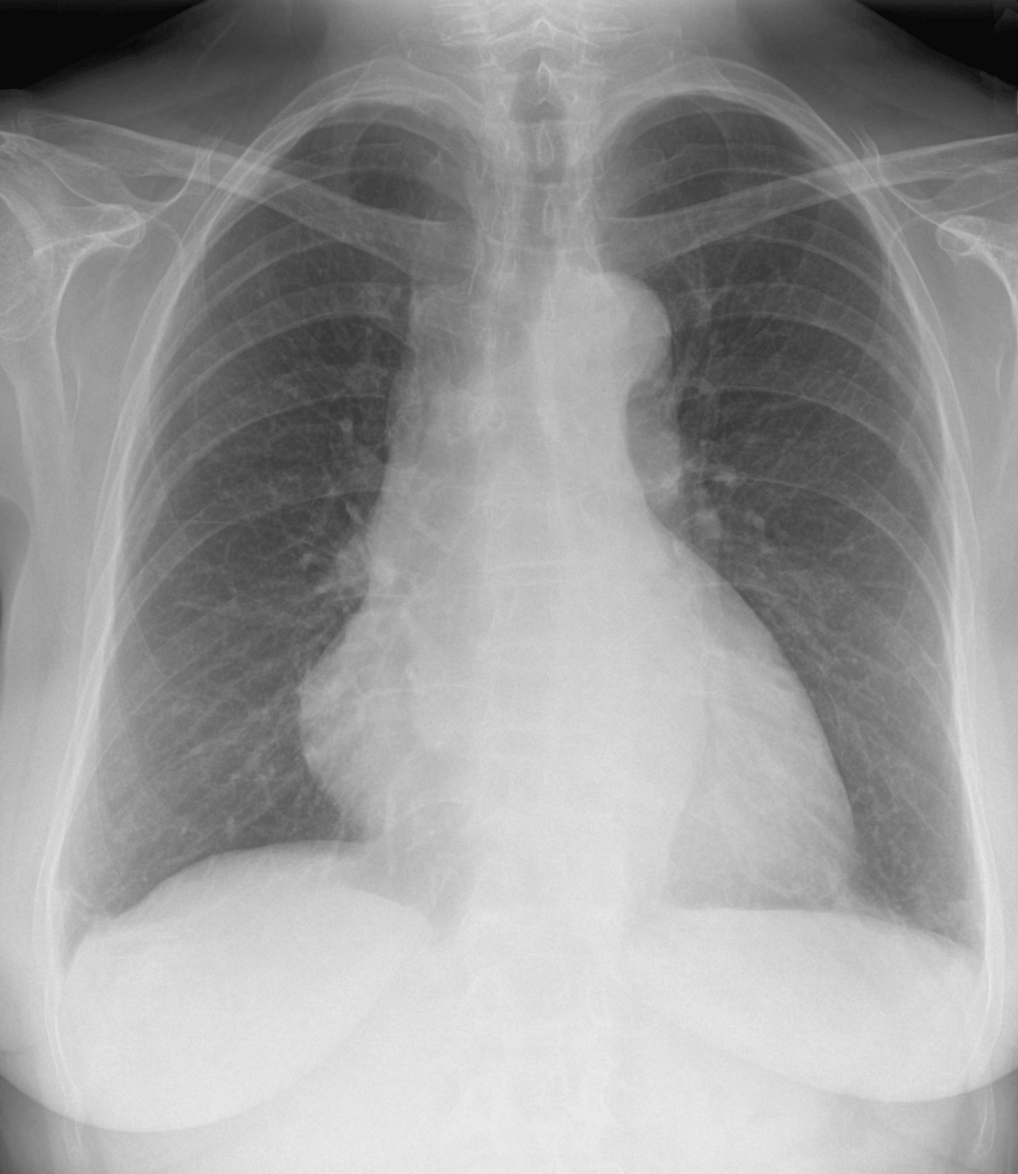
Chest X-ray after 2 weeks showing resolution of pleural effusion

After the initial workup, our differential diagnoses were lower respiratory tract infection and PCIS. She was initially started on intravenous amoxicillin/clavulanic acid to cover lower respiratory tract infection. Blood culture was negative. Diagnostic pleural tap showed exudative effusion according to Light’s criteria. Bacteriological culture and cytology for malignant cells were negative. After five days of antibiotics, she showed no signs of improvement, and there was no resolution of pleural effusion on chest X-ray. Computed tomography (CT) of the chest showed persistent bilateral pleural and pericardial effusion without any suspicious nodule or consolidation. Procalcitonin, autoimmune screen including antinuclear antibody (ANA), antineutrophil cytoplasmic antibody (ANCA), anti-cyclic citrullinated peptide (anti-CCP) antibody, and rheumatoid factor (RF) antibody were negative. Due to her recent atrial flutter ablation, PCIS was considered. In view of this, she was started on a short course of oral prednisolone at 0.5 mg/kg/day with oral colchicine 0.5 mg twice a day. As the patient was not in pain and infection was ruled out, steroid was chosen over non-steroidal anti-inflammatory drugs (NSAIDs). After a few days, the patient showed clinical improvement in terms of shortness of breath, and CRP markedly declined to 55 mg/L from 258 mg/L on admission. A repeat chest X-ray showed partial resolution of pleural effusion. A repeat echocardiogram showed pericardial effusion reduced to 0.5 cm around the base. The patient was discharged and followed up at the outpatient clinic after two weeks. CRP was reduced to <0.5mg/L, and a repeat chest X-ray (Figure 3) showed complete resolution of her pleural effusion. Similarly, the bedside echocardiogram showed no evidence of pericardial effusion. Steroid was slowly tapered over six weeks, and she was advised to continue colchicine 0.5 mg twice a day for three months in total.

## Discussion

PCIS from cardiac catheter ablation is not so commonly reported. A systematic review by Li et al. looking at PCIS after catheter ablation reports that PCIS is more likely to complicate atrial fibrillation/atrial flutter ablation (71.4%) compared to other ablation procedures, such as AVRT (9.5%), AVN (9.5%), AVNRT (4.8%), and VT ablation (4.8%) [8]. This likely reflects the larger area of myocardial injury caused by atrial fibrillation/flutter ablation compared to other procedures and subsequent autoimmune response, as suggested by Koller et al. [9]. This may also reflect a thinner atrial wall and a higher likelihood of pericardial injury compared to ventricular ablation.

The symptoms range from most commonly reported fever, pleuritic chest pain, and pleural and pericardial effusion to uncommon features, such as pulmonary infiltrate, massive pleural effusion, and even fatal cardiac tamponade [8]. A proposed criterion requires at least two of the following to be present to diagnose PCIS following cardiac injury: (1) fever without an alternative cause, (2) pleuritic chest pain, (3) pleural or pericardial rub, (4) Pleural or pericardial effusion, and (5) elevated CRP [10]. Our case had most of the common presentations of PCIS, such as pleural effusion, pericardial effusion, fever, and raised inflammatory markers. However, the lack of chest pain and typical ECG features of pericarditis presented a unique challenge, and we had to consider chest infection and heart failure as differential diagnoses. These two differentials were subsequently ruled out due to a lack of response to antibiotics and negative NT-proBNP, respectively. Another possibility would be cardiac perforation following ablation, but this was ruled out by post-procedure echocardiography. In addition, the two-week latency would favor PCIS over other differentials. 

NSAIDs and aspirin are considered first-line treatment for PCIS, with corticosteroids being reserved as second-line treatment for PCIS when NSAIDs or aspirin are contraindicated. Although corticosteroids produce rapid control of symptoms, they favor chronicity and recurrence. Steroids should be initiated once infection is ruled out, usually starting at 0.25-0.5 mg/kg/day. It should only be tapered once inflammation is controlled, and slow tapering is recommended to prevent recurrence [10]. In our case, corticosteroid was deemed suitable as infection was ruled out. Moreover, since the patient did not have any pain symptoms, a short course of steroids was preferred over NSAIDs. Colchicine is also advised for PCIS to prevent recurrence.

## Conclusions

PCIS after catheter ablation of arrhythmias can present as a diagnostic challenge. It is not commonly reported and may present with atypical features. Depending on the presentation, the differentials may include heart failure, chest infection, and cardiac perforation. We have illustrated how a systematic exclusion of differentials and an open-minded approach can lead to this diagnosis, allowing prompt treatment and better patient outcomes.
